# Cervical arthroplasty using ProDisc-C Case Report

**Published:** 2013-03-25

**Authors:** DA Nica, R Copaciu

**Affiliations:** Neurosurgery Clinic, “Sf. Pantelimon" Emergency Hospital, Bucharest, Romania

**Keywords:** disc replacement, disc herniation, disc degeneration

## Abstract

Cervical disc replacement is an emerging motion-preserving technology in the surgical treatment of the cervical degenerative disc disorders used as an alternative to the classic interbody fusion. We present a case report of a patient diagnosed with C6-7 right disc herniation who underwent anterior discectomy and received a total disc replacement using ProDisc C artificial disc prosthesis.

## Introduction

The intervertebral disc is a fibro cartilaginous structure consisting of nucleus pulposus in the center which is surrounded by annulus fibrosus and respectively, the superior and inferior end plates. The main purpose of the disc is to absorb shocks but also to allow movement between the vertebral bodies. The disc degeneration is responsible for the nucleus pulposus herniation and it appears as a result of a complex interaction between biological, genetic and biomechanical factors [**1**]. For many years, anterior decompression and discectomy with interbody fusion using an internal fixation device was the standard treatment for cervical disc herniations [**2**]. Because of the concern of adjacent level degeneration, after the fusion of a mobile vertebral segment [3,4], mobile disc prosthesis has been developed, which mimics the natural movement of the spine allowing mobility on all axes. 

**Fig. 1:**
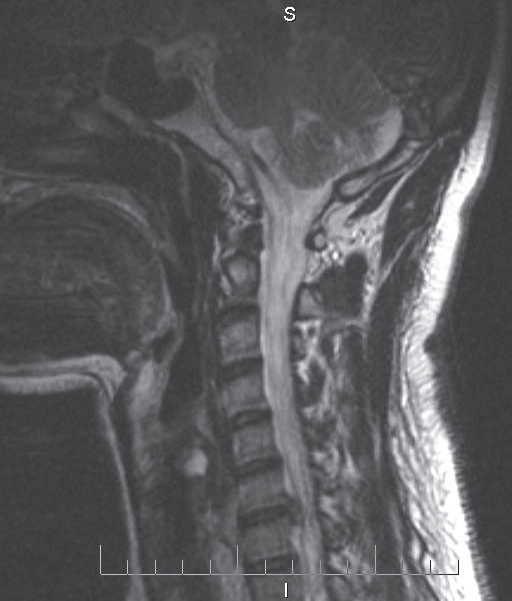
Cervical MRI showing C6-7 right disc herniation

**Fig. 2:**
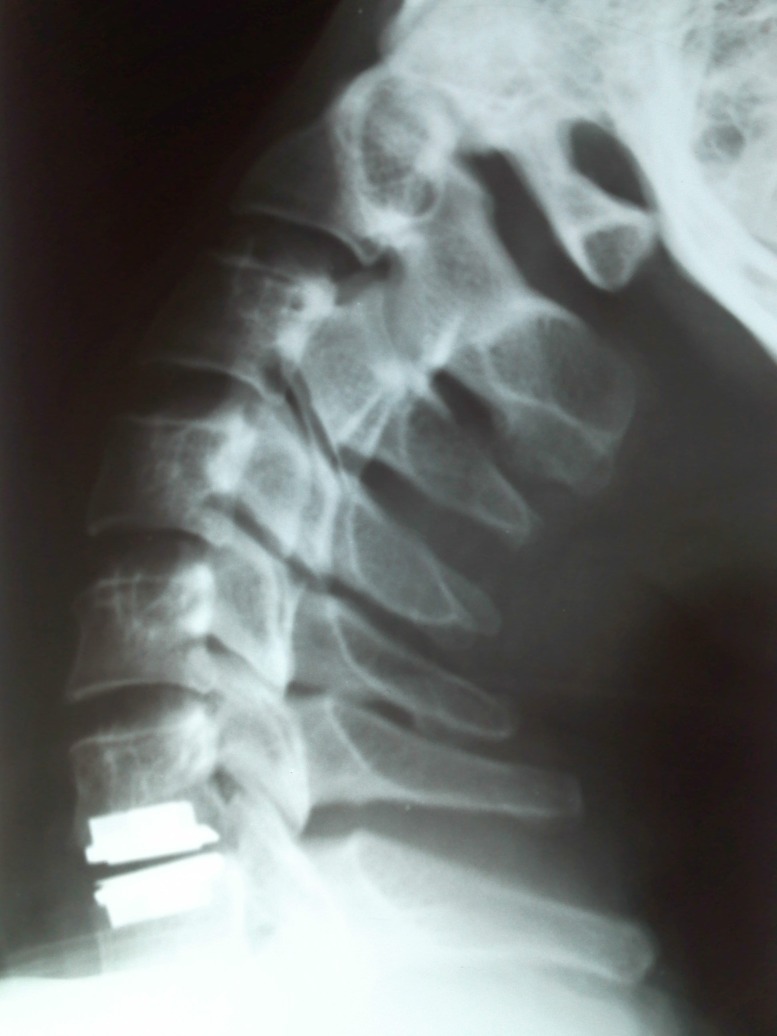
Postoperative cervical x-ray, correct position of Prodisc C prosthesis

 A 34-year-old male patient came to our Clinic reporting cervical, right shoulder and right arm pain. The pain lasted for 4 weeks and after an intense physical effort, symptoms worsened during the last 5 days before coming to the Clinic. He also reported that he received a treatment with non-steroidal anti-inflammatory drugs. The neurological examination found a reduction in the mobility of the cervical spine because of the pain, right arm hypoesthesia in C7 nerve territory accompanied by motor deficit also in the right C7 nerve territory. The patient was committed and a cervical spine MRI was performed. The MRI showed a right C6-7 disc herniation compressing the C7 nerve root (**Fig. 1**). Considering the clinical symptoms and the MRI result, the patient underwent surgery. Anterior approach was adopted to expose the intervertebral disc followed by discectomy with the decompression of the C7 nerve root. After complete removal of the nucleus pulposus and annulus fibrosus along with the adjacent terminal plates, under 2,5x magnification, maintaining the bony integrity of the body of vertebra, it was opted for a disc replacement using a Prodisc C prosthesis. Prodisc C has a semi-constrained design that provides a fixed center of rotation and it is designed to allow controlled dynamic motion through the physiological range of motion and to prevent pure translational motion to theoretically protect the facets from excessive shear loading. Intraoperative X-ray was done to confirm the correct positioning of the prosthesis. Post-operative, the patient’s neurological symptoms improved with no cervical, shoulder or arm pain. He was immobilized with a cervical collar for 2 weeks. One week after surgery cervical X-ray showed the disc prosthesis maintaining its original position (**Fig. 2**). After kinetic therapy, the patient’s motor deficit improved regaining full function one month after surgery. 

## Discussions

Anterior cervical discectomy and decompression in combination with interbody fusion using a bone graft is a classic method for treating cervical degenerative disease. Studies show a possible degeneration of adjacent discs after fusion, due to the increase in motion and pressure of the adjacent discs [4,5] making mobile cervical prosthetics a much needed alternative. 

Unlike interbody fusion using bone grafts, cervical arthroplasty provides stability and very good fixation, Prodisc C having endplate central keels with titanium coating for promoting bone ingrowth, thus preventing possible implant migration with the need of a reintervention. 

 There are multiple restrictions when considering using such prosthesis, one of them being the cost. Even if it appears to be expensive, considering the fact that it might help prevent further adjacent disc degeneration with possible further surgical intervention for decompression at those levels, it could prove economic by saving the second or even third surgery costs.

## Conclusions

In comparison with the interbody fusion, which reduces de mobility of the cervical column, being subject to other possible complications, Prodisc C prosthesis allows a good mobility, equivalent to the natural mobility of the cervical column, functioning also as a shock absorber. 

 Using this type of prosthesis helps prevent adjacent discs degenerations followed by surgical interventions, making its cost efficient despite the apparent high price.
